# Experiences of Racial Discrimination: Qualitative Findings from Injured New Zealand Migrants

**DOI:** 10.1007/s10903-026-01871-6

**Published:** 2026-02-06

**Authors:** Kelly Radka, Sarah Derrett, Emma Wyeth

**Affiliations:** 1 Ngāi Tahu Māori Health Research Unit, University of Otago, Dunedin, New Zealand; 2 Ngāi Tahu Research Centre, University of Canterbury, Christchurch, New Zealand

**Keywords:** Migrants, Racism, Racial discrimination, Structural racism, Injury, New Zealand

## Abstract

Racism is a recognised determinant of health internationally and in New Zealand (NZ). Racism is instantiated through racial discrimination, including discriminatory attitudes and practices. Associations between racial discrimination and unfavourable health-related experiences and outcomes have been reported for specific groups in NZ, including for Māori (the Indigenous people of NZ) and for those of Asian and Pacific ethnicities. However, less appears to be known about NZ migrants’ (those defined as non-NZ born) experiences of racial discrimination. This paper reports injured NZ migrants’ experiences of racial discrimination through qualitative analyses of free-text responses to a set of racism-related questions within the ‘Prospective Outcomes of Injury Study 10 Years On’ project. Participants reported experiences of racial discrimination in a range of contexts, including at work, in public, in housing-related contexts(in neighbourhoods and whilst seeking housing) and in health settings. Experiences comprised physical violence and intimidatingbehaviour, racially motivated verbal abuse, discrimination based on appearance or communication, stereotyping, and having their “NewZealand-ness” challenged. Reported impacts of racial discrimination included reduced work opportunities and feeling ‘othered.’ Migrants’ experiences of racial discrimination (across a range of contexts) are potentially underpinned by structural racism in NZsociety. This can affect migrants’ health and well-being through compromised socio-economic opportunities (and by corollary, reduced access to resources) and potentially, lower engagement with societal supports and services. We call for similar research internationally to investigate potential links between experiences and impacts of racial discrimination and future engagement with societal supports, including health and injury services.

## Background

Racism refers to societal systems and ideologies which create and perpetuate inequities in power, access to resources and opportunities for certain groups [1, 2]. Racism is a recognised determinant of health internationally [1, 3, 4] and in New Zealand (NZ) [5–7]. Experiencing racial discrimination (the mechanism by which racism is instantiated/enacted) including discriminatory attitudes and practices [5, 8] can negatively impact health and well-being as a consequence of marginalisation, exclusion, disadvantage and conflict [1].

Racial discrimination is produced through mutually reinforcing patterns and practices at individual/interpersonal, institutional and structural levels [8–11] in a range of sectors. Understanding the nature and impact of racial discrimination *within* a specific sector (e.g., housing, employment and the health sector) requires investigation into the broader social contexts within which these are situated, including how these operate *alongside* other sectors [2, 3].

Racial discrimination is not experienced evenly by all ethnic groups in NZ [2, 12], with Māori (the Indigenous people of NZ) [13], Pacific, and Asian ethnicities, reporting greater incidence of racism than those of European ethnicity [6]. Links between racial discrimination and poor health have been well established in NZ, including for Māori, who (grounded in the legacy of colonialism [13]) experience a range of health inequities, including shorter lifespans than non-Māori [14]. Less appears to be known regarding the experiences of NZ migrants [7] (defined as non-NZ born).

Migrants comprise 28% of the NZ population [15] and have been found to experience racial discrimination in a range of settings. At work, migrant workers are more likely to report racial discrimination than NZ-born workers [16]; including related to appearance and religious clothing [17], perceived cultural differences [18], accent, and English language proficiency [18–20]. Professional discrimination may occur through non-recognition of country-of-origin qualifications, and perceptions migrants are differently [and, less] qualified [19–21]. Further, employers and the State may characterise migrants of specific nationalities as ‘good’ workers, thus tacitly favouring the contributions of some migrants over others [22, 23].

Structural racism within property markets can shape access to housing. Migrants may be characterised as ‘risky’ tenants, based on assumptions of low socio-economic and precarious employment status, and cultural/ethnic stereotypes [24]. Property market ‘gatekeepers’ can marginalise migrant tenants through tacit socio-cultural restrictions on housing-related practices [24, 25], including disinclination to rent property to those of certain ethnicities [24].

During health interactions, NZ migrant service users report discriminatory attitudes [26] from health professionals [27–29], including discrimination based on perceived English language proficiency [30], and dismissive attitudes when expressing health concerns, or describing country of origin health services and practices [31]. Migrants may be stigmatised as “difficult” patients, including regarding disposition to treatment and pain tolerance [32]. More broadly, during the COVID-19 pandemic, migrants were framed as contributing to the spread of disease and as detracting from services available for “Kiwis” [33, 34] [colloquial term for a New Zealander].

Previous investigations of injury experiences in NZ have largely focused on ethnicity as a variable to be considered, with findings for Māori [35–38], Pacific peoples [39], and Asian ethnicities [40]. Findings for Asian ethnicities include reports of injuries due to racially motivated assaults [41], and discrimination during interactions with the Accident Compensation Corporation (ACC) (NZ’s national no-fault injury compensation scheme) [42] based on perceived cultural differences and worldviews, English language proficiency, and negative attitudes of staff [40]. Such discrimination can contribute to access barriers, along with lower satisfaction with services when they are accessed [40].

Quantitively, injured NZ migrants have been found to experience higher risk of disability than NZ-born counterparts at three months post-injury [43]. To the best of our knowledge, few empirical studies have qualitatively investigated the lived experiences (including experiences of racial discrimination) of injured NZ migrants [44].

The aim of this paper was to report findings regarding the ‘‘who, what and where” [45] of injured NZ migrants’ experiences of racial discrimination across a range of settings (including health care, employment and housing) through qualitative descriptive analysis of free-text responses from interviews undertaken with injured migrants who participated in the longitudinal injury cohort study- the Prospective Outcomes of Injury Study (POIS) [46], and its follow-up, the Prospective Outcomes of Injury Study 10 years on (POIS-10) [47].

## Methods

### Study Context and Participants

POIS recruited 2856 injured New Zealanders between 2007 and 2009, from five regions of NZ, with the aim of investigating factors leading to health and disability post- injury [46], for adults who had an ACC entitlement claim injury. POIS participants (aged 18–64 years) participated in telephone interviews, approximately 3, 12 and 24-months post-injury [48]. In 2020, POIS-10 (10 years after their last POIS interview) aimed to investigate longer-term outcomes of injury for the POIS cohort [47]. POIS and POIS-10 received ethical approval from the Health and Disability Ethics Committee New Zealand (MEC/07/07/093/AM07).

Questions related to experiences of racial discrimination (adapted from the NZ Health Survey 2020/2021 [49]) in the POIS-10 interview-administered questionnaire, asked participants if they had ever been: (1) A victim of an ethnically motivated attack (verbal or physical abuse to you or your property) in NZ, (2) Treated unfairly by a health professional because of your ethnicity in NZ, (3) Treated unfairly at work (including being refused a job) because of your ethnicity in NZ, and (4) Treated unfairly when renting or buying housing because of your ethnicity in NZ? Participants were then asked an open-ended question: *“Can you tell me what you have experienced?”* with their verbatim responses entered by the interviewer. This paper presents findings from analyses of these free-text responses from non-NZ born participants responding to this question in the POIS-10 interview.

## Data Collection

Trained interviewers conducted POIS-10 [47] telephone interviews; responses were entered into REDCap [50]. Responses were recorded in the form of verbatim quotes, with some interviewers recording summarised comments. Responses ranged in length from a few sentences to longer (approximately 15 sentences). The responses were imported into *NVivo* [51] with a record for each participant.

## Analysis

To remain close to the data as reported [52], a qualitative descriptive [53] approach, using thematic Qualitative Text Analysis, was a pragmatic choice. Following steps outlined by Kuckartz [54] careful reading of each participant record was undertaken, with annotations made, including initial descriptive comments and reflections. Next, initial open codes were generated from participant records, according to three developed “topical categories” derived directly from the research aim [54]. These included: (1) the ‘where’, comprising descriptive details regarding the contexts of reported experiences, (2) the ‘who’, comprising descriptive characteristics of the reported perpetrators of the racial discrimination, and (3) the ‘what’, consisting of the nature of reported racial discrimination.

Topical categories were then refined to include sub-categories. At this stage, an additional category was generated based on reported experiences of the impacts of racial discrimination, as participants frequently highlighted this in their responses. Subsequently, a final round of coding was undertaken, wherein all data were coded to the refined category system, consisting of four developed topical categories and generated sub-categories. These are presented below, following an overview of participant characteristics.

## Results

### Participant Characteristics

Within POIS, 677 POIS participants were ‘non-NZ born’, of whom 512 completed an interview at 24 months post injury. Of these, 467 did not decline future contact for a follow-up interview. Ten years later, of the 467, 104 were uncontactable, 34 declined an interview when contacted, four were known to have died, two had communication difficulties precluding participation; 323 (90%) of those who were contactable, participated in a POIS-10 interview [43].

Of the 323 who completed a POIS-10 interview, 105 provided a free-text response to the question *“Can you tell me what you have experienced?”* Of these, 44 were female and 61 male. Their mean age (at the time of injury) was 45 years. Participants were born in a range of countries, including countries in Africa, Asia, Europe, the Middle East, North and South America, and Pacific Island nations.

### Developed Topical Categories and Sub-Categories

**Fig. 1:**
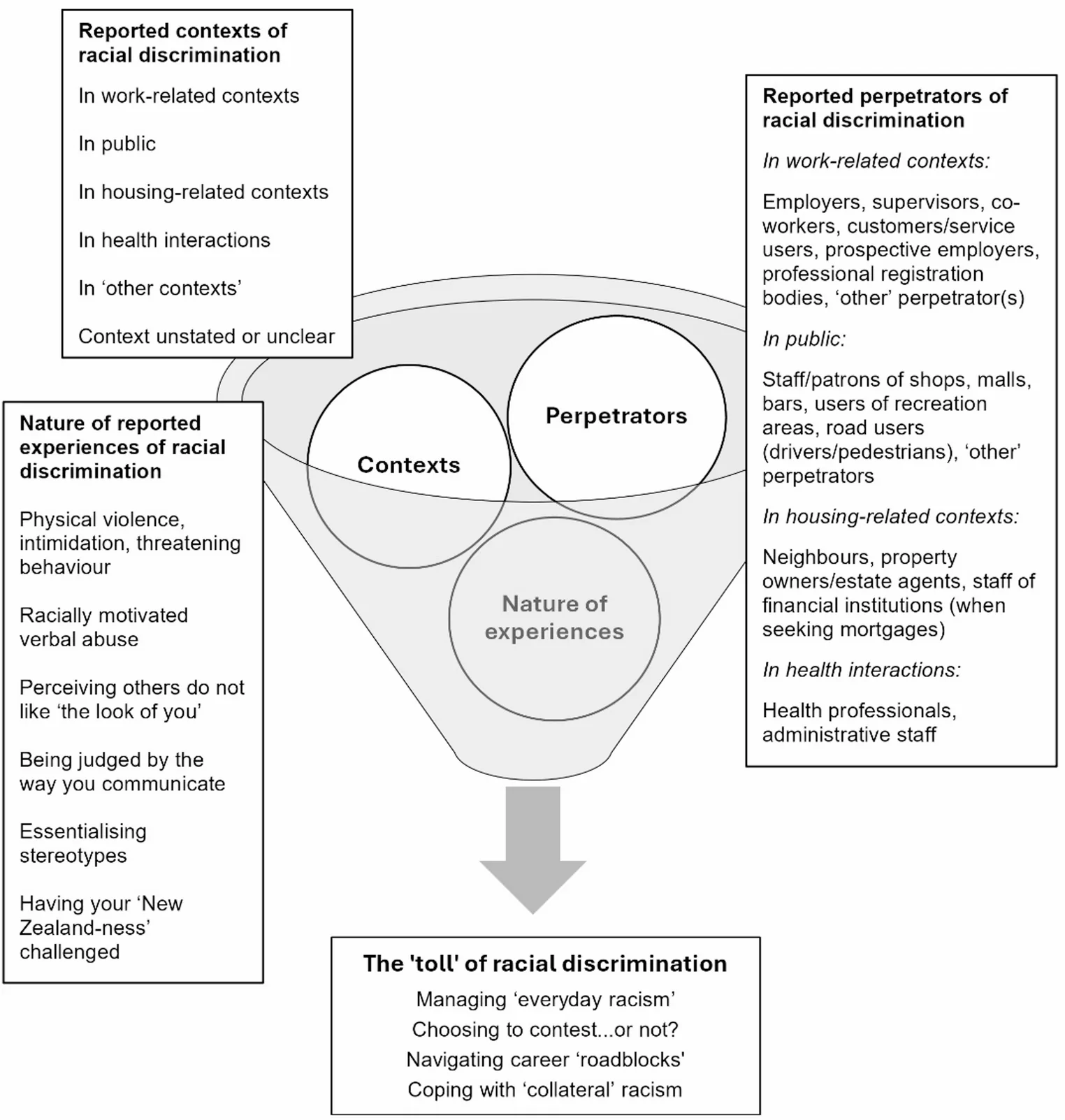
Developed topical categories

Developed topical categories and sub-categories are presented in Fig. 1. Following the figure, these are elucidated, accompanied by illustrative quotes from participants (including self-reported country of birth) highlighting the essence of each.

## The Contexts and Perpetrators of Racial Discrimination

Participants described experiences of racial discrimination in a range of (and in some cases, multiple) contexts, with these reported in Table 1. The majority of these experiences occurred in work-related settings, followed by ‘in public’ (in a range of contexts) and in participants’ neighbourhoods.

**Table 1:** Contexts and perpetrators of Racial discrimination reported by POIS-10 injured migrant participants (*n* = 105)

Reported contexts of experiences of racial discrimination	n*
Work-related, including in interactions with: • Employers/supervisors • Co-workers • Customers/service users • Prospective employers • Professional registration bodies • Other perpetrator(s) at work	38
In public (in a range of settings), including in interactions with: • Staff/patrons of shops, shopping malls and pubs • Users of recreation areas • Road users (including drivers and pedestrians) • Other perpetrator(s) in public	23
In housing-related contexts, including in interactions with: • Neighbours • Property owners/agents • Staff of financial institutions (when seeking mortgages)	11
In health interactions with: • Health professionals • Administrative staff at health services	3
In ‘other’ contexts	8
Context unstated or unclear	36

*Some participants described experiencing racial discrimination in more than one context and by more than one perpetrator

## The Nature of Experiences of Racial Discrimination

Some participants described experiences of racial discrimination instantiated through physical violence, and intimidating/threatening behaviour, *“…physical abuse- throwing stuff at me…magazine…whatever is in their hands…screaming and shouting at me-spitting*,* throwing water…adults 25 plus [of age] white male/female…at work and socially” (P53*,* Fiji)*, whilst approximately half had experienced racially motivated verbal abuse. Others reported more ‘opaque’ discrimination based on responses to their appearance; perceiving others did not like ‘the look of them’ *(P5 & 92*,* Singapore*,* P102*,* Bahrain).*

Participants experienced racial discrimination during everyday verbal exchanges, including discrimination based on ‘non-local’ accents, *“…people pretending not to understand my accent*,* having jokes about me” (P34*,* England)* and their communication style (including tone and register), *“…with my ex-partner of 7 years-we were renting. Because he was [nationality]*,* he talks loud…the surrounding neighbours thought we were fighting-the body corp [corporate] landlord did not renew our contract to stay.” (P73*,* Fiji)* Others faced assumptions of low English language proficiency (regardless of actual proficiency).

Other participants reported racial discrimination via stereotypes, including assumptions of low socio-economic status and criminality. Further, a smaller number experienced racial discrimination through unwarranted scrutiny or being made to wait at appointments for longer, *“…other people would come in after me and go in first-happened more than once.” (P38*,* Philippines)* Others reported being made to feel they did not belong in NZ, including through having their status as New Zealanders challenged, *“First thing is*,* asking why I live here and not in my home country?” (P61*,* Fiji)* and for some, being ‘shamed and blamed’ for adverse global geopolitical events. Table 2 presents further examples of reported experiences, including illustrative verbatim quotes.

**Table 2:** Illustrative quotes regarding the nature of experiences of racial discrimination

Nature of experiences of racial discrimination	Illustrative quotes	
Physical violence, intimidation, threatening behaviour	*…overheard someone say that they wished all the Americans had died in 911… (P63*,* United States)*	*When I tried to get past him in the corridor at work-I didn’t know him. He didn’t budge… (P8*,* England)*
Racially motivated verbal abuse	*Every day in my [name of workplace] I have racial comments. If people say they don’t have enough money to buy something*,* I say how about you buy it in a smaller quantity? I get called a ‘F------ [expletive] Indian’. (P58*,* India)*	*He [co-worker] passed comment about my parking one day-called me a ‘ching chong’. (P92*,* Singapore)*
Others not liking ‘the look of you’	*I applied for a [identifying details removed] job and the company wrote and said I was just what we are looking for*,* come for an interview. When I got there*,* I waited and waited at reception and the interviewer*,* she came and she looked at me*,* then she said*,* ‘I’m sorry the job has been taken.’ I knew that she didn’t want me because of my look. (P5*,* Singapore)*	*I was working in [name of workplace]*,* coming into work in my cultural clothes and changing to work uniform*,* I got picked on because I was wearing my cultural and religious clothes. (P61*,* Fiji)*
Being judged by the way you communicate	…*people feel uncomfortable around us if we are being more vocal and talk about what we are doing. (P94*,* Argentina)*	*Just comments*,* what I look like and the way I talk. (P105*,* Malaysia)*
Facing essentialising stereotypes	*When I go to buy things*,* they think I can’t afford it. (P88*,* Tonga)*	*…looked at as though I will be violent. (P61*,* Fiji)*
Having your ‘New Zealand-ness’ challenged	*Someone kept taunting me for the words I was using. They didn’t understand I was using words correctly. Because of my accent*,* they said I wasn’t a New Zealander. (P9*,* Scotland)*	*…I come into contact with lots of different people in my work. They say that*,* as an immigrant*,* I shouldn’t be here. (P104*,* Netherlands)*

### The ‘Toll’ of Experiencing Racial Discrimination

In sharing their experiences of racial discrimination, some participants also shared the impacts or ‘toll’ of these experiences. Firstly, some ‘normalised’ these experiences, characterising them as unpleasant, yet relatively routine occurrences, *“Unfortunately*,* at the wrong place and the wrong time…” (P26*,* Zimbabwe)* Participants described deploying strategies to manage ‘everyday racism’ including ‘playing down’ the impact of such incidences, *“…often received verbal abuse from teenagers-they like to drive around and if they see someone who looks different*,* they scream at them. Doesn’t really bother me too much…” (P92*,* Singapore)*.

Whilst some participants normalised experiences of racial discrimination, others reflected on the insidiousness of these, including a lasting impact, and contributing to an added cognitive ‘load’: *“…verbal comments at work all the time in NZ from workmates and some [service users] …Work mates bothered me for 10 years. Bullying. [The situation was] …better in Australia.” (P38*,* Philippines)* Some coped with racial discrimination through intentional avoidance of confrontation with perpetrators, *“…stopped by police- don’t argue… and where are you from” (P17*,* Egypt)* whilst others actively contested the racial discrimination and challenged the perpetrators.

A range of experiences of workplace racial discrimination were reported, with some perceiving these contributed obstacles to career progression. This included colleagues expressing resentment if participants were perceived to be in a position of privilege in the workplace. Alongside this, others had their skills challenged in the workplace, *“…other cases being looked at like a stupid person or questioned more than the average person…” (P61*,* Fiji)* Crucially, although some perceived they were treated differently in the workplace, others found it challenging to definitively attribute negative experiences to racial discrimination, *“It’s difficult to know. At work*,* promotion-related…I cannot be sure if ethnically motivated or not…under-representation in management is obvious” (P35*,* Malaysia)* and:

“I never got promoted and my boss wasn’t happy when I got my injury in 2008…she [work supervisor] made it difficult and I felt the pressure. I got an infection at work and thought I would lose my finger because of work. I think that there was some cultural/race differences. I’ve often thought about it.” *(P68*,* Sri Lanka)*.

Such beliefs were persistent and difficult to ignore, contributing to the highlighted insidiousness of experiences.

Unsurprisingly, directly experiencing racial discrimination had a negative and lasting impact on participants. Further, participants were affected by racial discrimination directed towards those close to them, including family members, loved ones and colleagues. Experiences of ‘collateral’ racial discrimination had intertwining practical and affective consequences, including being turned down for rental housing and mortgages, and feeling uncomfortable at work, perceiving racial discrimination as ingrained in the work culture.

The majority of those offering a free-text comment regarding experiences of racial discrimination, described profoundly negative experiences. Nevertheless, a small number of participants expressed a counter-narrative, in that, they expressed a sense of belonging in NZ, including that they identified as ‘Kiwis.’ Others, in addition to a sense of belonging, expressed gratitude, *“I’m glad that I live here in New Zealand- it is the best place to live*,* and I am feeling like a New Zealander.” (P59*,* Fiji)* Further, some felt attitudes (and tolerance) of racial discrimination were changing for the better, *“When I came to this country in 2002*,* it was different*,* people didn’t really accept us*,* but nowadays things are better*,* there is more acceptance in society.” (P58*,* India)* Such comments indicate negative experiences of racial discrimination were not reported universally. Nevertheless, as some participant records included both positive and negative sentiments, it is unclear whether participants were satisfied with the extent and nature of perceived changes for the better and further, whether expressing optimism might be a way of coping with negative experiences. Table 3 presents further examples of the impacts of experiencing racial discrimination, accompanied by verbatim quotes from participants.

**Table 3:** Illustrative quotes regarding the impacts of racial discrimination

Impacts of experiencing racial discrimination	Illustrative quotes	
Normalising the discrimination: Managing ‘everyday racism’	*I get verbal and threatening abuse on the [name of work area] when I’m at work because I’m Indian. It happens on and off. (P91*,* India)*	*…if you walk down [street name] late at night you will meet some drunken idiots mouthing off. I am physically fit and not intimidated by it. (P33*,* China)*
Choosing to contest (or not)?	*There’s also been comments when I have been a customer*,* making comments about my ethnicity. I asked why I was treated differently. (P61*,* Fiji)*	*I went to the WINZ [Work & Income] office. I wanted to speak to them about my ex-wife*,* the receptionist was rude to me. I looked at her and walked away*,* otherwise I would have said something I should not say. (P29*,* South Africa)*
Contributing to career ‘roadblocks’	*…So being told that they [employers] recognise they didn’t have an ethnically diverse group*,* that they were reluctant to employ somebody of Indian origin*,* as they were concerned about both my accent and origin. (P19*,* England)*	*…they wouldn’t even consider me for promotion to team leader because I’m Dutch. Dutch people are known to have strong opinions and say what they think - they wouldn’t even consider my application. (P99*,* Netherlands)*
Coping with ‘collateral racism’	*…at work*,* being in the [occupational sector] there’s a variety of people and sometimes other teams that say stuff. Some of it may not directly be at me but still makes me feel uncomfortable. (P57*,* Tonga)*	*My wife is from Tonga and we have been turned down for a house to rent because of this.* *(P48*,* Australia)*

## Discussion

Our investigation reports a range of experiences of racial discrimination of injured NZ migrants, including racially motivated verbal abuse and socio-cultural stereotypes, with a smaller number of participants reporting racially motivated physical violence, intimidation and threatening behaviour. In NZ, associations have been established between racism and unmet healthcare needs, and lower satisfaction/less favourable experiences in access to healthcare for different ethnicities, with calls for investigation into the mechanisms through which these occur [6]. Our findings (in alignment with NZ Human Rights Commission findings [55]) highlight racial discrimination experienced by migrants is enacted through different (and often multiple) mechanisms, including based on race, ethnicity, appearance, communication style, perceived English language proficiency, and through having their belonging in NZ (as migrants) both overtly and indirectly contested.

Our findings regarding the toll of racial discrimination, suggest migrants’ experiences of individual/interpersonal racism may be grounded in wider structural racism. At work, participants reported having their skills contested by others, under-representation of others ‘like them’ in workplaces, and compromised pathways to promotion. Such experiences can contribute to reduced economic opportunities and advantages, which, in turn, can shape access to health-promoting resources and opportunities [56].

Participants highlighted experiences of being ‘othered.’ This occurs through attitudes/practices which establish and perpetuate boundaries between individuals and groups in society, resulting in the privileging (and marginalisation) of some over others [57]. Such experiences can detract from feeling connected to (and safe in) wider society, thereby negatively affecting overall wellbeing [58], along with potentially contributing to reduced trust in societal supports/systems. Migrants’ inclusion in communities can shape their well-being, through access to community and societal resources and supports [26]. Although othering may be perpetuated by individuals, this may be indicative of wider societal perceptions and messaging surrounding migrants and their contributions [55], including the extent to which they ‘belong’ in NZ society; aspects requiring further investigation.

Participants were recruited into POIS in 2007–2009 [46], with questions regarding their experiences of racial discrimination at the POIS-10 interview (in 2020) [47]. This meant participants were interviewed by trained interviewers at multiple timepoints post-injury (since 2007–2009), fostering rapport [48] and willingness to share a range of experiences, including experiences and impacts of racial discrimination. Nevertheless, investigation into the experiences of more recently arrived migrants (e.g., post-2020) warrants investigation.

We have identified high-level descriptive findings regarding the reported perpetrators of racial discrimination. Further research is required (in NZ and internationally) to elucidate the potential role of intercultural/inter-ethnic tensions and lateral violence [7] in migrants’ experiences of racial discrimination and the impacts of this on access to societal resources.

## Conclusion

This research presents a snapshot of experiences of racial discrimination across a range of settings of NZ migrants with a shared health-related experience, the experience of an injury. Injuries are (unfortunately) a common experience in NZ, with over two million injury claims registered with ACC in 2023/2024 [42], from a population of 5,356,700 (2024) [59].

Our findings indicate migrants experience racial discrimination in a range of contexts in NZ, with these enacted through a range of mechanisms, and potentially, underpinned by aspects of structural racism. These experiences have direct (and negative) consequences for migrants, including contributing to compromised work opportunities (and by corollary, socio-economic opportunities) which could negatively affect migrants’ access to various resources to support their well-being. Further, migrants’ health and well-being may be affected by the extent to which they feel a sense of belonging (and are made to feel they belong) in NZ society, as this could contribute to engagement (or disengagement) with societal resources and services, including, potentially, health and injury services. Such services should be cognisant of this, and take action to mitigate this potential, including through targeted staff training and outreach to migrant communities.

We advocate for further research internationally, including: (a) Similar investigations in other countries, including those with different health and injury services, (b) Studies drawing upon intentional intersectional approaches to assess how experiences of racial discrimination may be shaped by intersectional identities, including migration status/pathways, gender, ethnicity, proficiency in the host country language and socio-economic status (as these, individually and collectively, may contribute to different experiences and outcomes) and, 3) Studies investigating potential links between experiences and impacts of racial discrimination and future engagement with societal supports, including health services (broadly) and injury services specifically.

## Data Availability

The datasets generated and/or analysed for this paper are not publicly available due to ethical constraints and potentially sensitive and identifiable data. If anyone is interested in pursuing collaborative research or discussing data sharing opportunities, please contact the corresponding author.
